# (1S*,2S*,4R*,5R*)-Cyclo­hexane-1,2,4,5-tetra­carb­oxy­lic acid

**DOI:** 10.1107/S1600536813033795

**Published:** 2013-12-21

**Authors:** Akira Uchida, Masatoshi Hasegawa, Shinya Yamaguchi, Eiichiro Takezawa, Atsushi Ishikawa, Takashi Kagayama

**Affiliations:** a Department of Biomolecular Science, Faculty of Science, Toho University, 2-2-1 Miyama, Funabashi, Chiba 274-8510, Japan; b Department of Chemistry, Faculty of Science, Toho University, 2-2-1 Miyama, Funabashi, Chiba 274-8510, Japan; c Department of Research and Development, Gas Chemical Division, Iwatani Industrial Gases Corporation Ltd., 10 Otakasu-cho, Amagasaki, Hyogo 660-0842, Japan

## Abstract

The title compound, C_10_H_12_O_8_, a prospective raw material for colourless polyimides which are applied to electronic and microelectronic devices, lies about an inversion centre and the cyclo­hexane ring adopts a chair conformation. Two crystallographycally independent carb­oxy­lic acid groups on adjacent C atoms are in equatorial positions, resulting in a mutually *trans* conformation. In the crystal, O—H⋯O hydrogen bonds around an inversion centre and a threefold rotoinversion axis, respectively, form an inversion dimer with an *R*_2_^2^(8) motif and a trimer with an *R*_3_^3^(12) motif.

## Related literature   

For background to polyimides, see: Ando *et al.* (2010 ▶); Hasegawa *et al.* (2007 ▶, 2013 ▶); Hasegawa & Horie (2001 ▶). For related structures, see: Uchida *et al.* (2003 ▶, 2012 ▶).
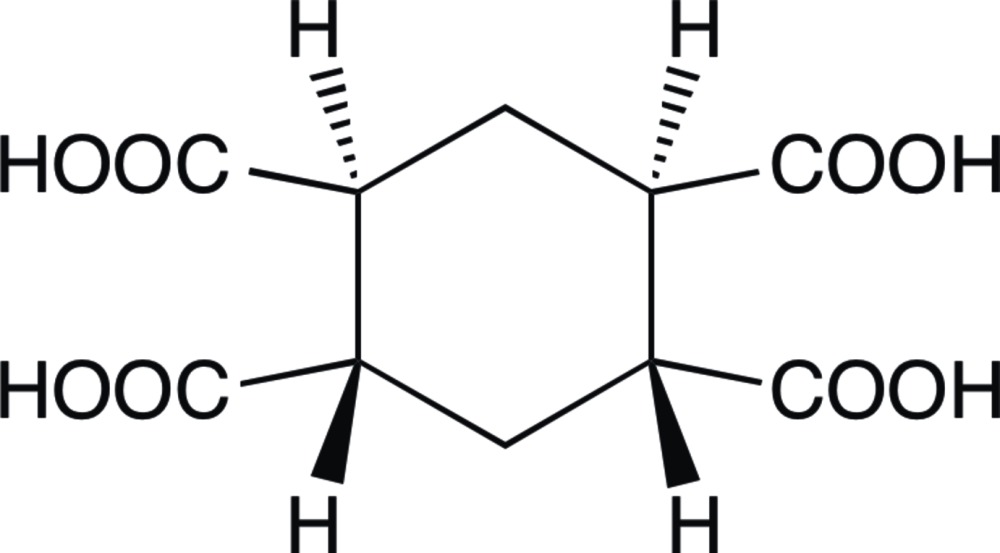


## Experimental   

### 

#### Crystal data  

C_10_H_12_O_8_
*M* *_r_* = 260.20
Trigonal, 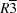

*a* = 17.6970 (6) Å
*c* = 9.5455 (6) Å
*V* = 2589.0 (2) Å^3^
*Z* = 9
Mo *K*α radiation
μ = 0.13 mm^−1^
*T* = 298 K
0.33 × 0.26 × 0.26 mm

#### Data collection  

Bruker APEXII CCD area-detector diffractometer
Absorption correction: multi-scan (*SADABS*; Sheldrick, 1996 ▶) *T*_min_ = 0.891, *T*_max_ = 0.966
6439 measured reflections
1653 independent reflections
1388 reflections with *I* > 2σ(*I*)
*R*_int_ = 0.019

#### Refinement  

*R*[*F* ^2^ > 2σ(*F* ^2^)] = 0.041
*wR*(*F* ^2^) = 0.119
*S* = 1.06
1653 reflections
90 parameters
H atoms treated by a mixture of independent and constrained refinement
Δρ_max_ = 0.38 e Å^−3^
Δρ_min_ = −0.21 e Å^−3^

### 

Data collection: *APEX2* (Bruker, 2007 ▶); cell refinement: *SAINT-Plus* (Bruker, 2007 ▶); data reduction: *SAINT-Plus*; program(s) used to solve structure: *SHELXS97* (Sheldrick, 2008 ▶); program(s) used to refine structure: *SHELXL2013* (Sheldrick, 2008 ▶); molecular graphics: *ORTEPIII* (Burnett & Johnson, 1996 ▶) and *PLATON* (Spek, 2009 ▶); software used to prepare material for publication: *SHELXL2013*.

## Supplementary Material

Crystal structure: contains datablock(s) global, I. DOI: 10.1107/S1600536813033795/is5327sup1.cif

Structure factors: contains datablock(s) I. DOI: 10.1107/S1600536813033795/is5327Isup2.hkl

Supporting information file. DOI: 10.1107/S1600536813033795/is5327Isup3.mol

Supporting information file. DOI: 10.1107/S1600536813033795/is5327Isup4.cml

Additional supporting information:  crystallographic information; 3D view; checkCIF report

## Figures and Tables

**Table 1:** Hydrogen-bond geometry (Å, °)

*D*—H⋯*A*	*D*—H	H⋯*A*	*D*⋯*A*	*D*—H⋯*A*
O2—H4⋯O1^i^	0.81 (3)	1.93 (3)	2.705 (2)	160 (3)
O4—H5⋯O3^ii^	0.94 (3)	1.70 (3)	2.632 (1)	176 (3)

Symmetry codes: (i) 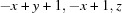; (ii) 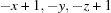.

**Fig. 1.**
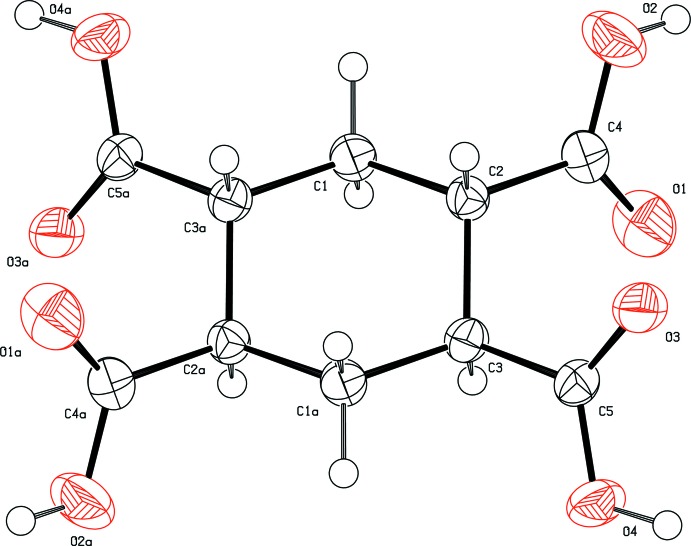
The molecular structure of the title compound, showing displacement ellipsoids at the 50% probability level. H atoms are represented by circles of arbitrary size.

**Fig. 2.**
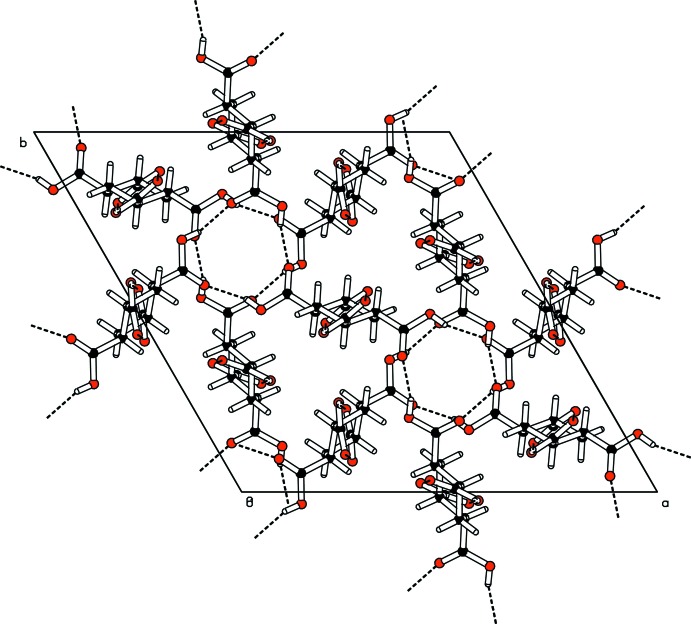
The packing of the title compound, viewed down the c axis,

## supplementary crystallographic information

### 1. Comment

Aromatic polyimides (PI) are one of the most important heat-resistant polymeric materials in various electronic applications for their reliable combined properties: considerably high glass transition temperatures (*T*_g_), non-flammability, and good dielectric and mechanical properties (Ando *et al.*, 2010). However, intensive coloration of conventional PI films, which arises from charge-transfer (CT) interactions (Hasegawa & Horie, 2001), often disturbs their applications as optical materials. A recent strong demand is to replace inorganic glass substrates in flat panel displays (300–700 µm thick) by plastic substrates (< 100 µm thick), thereby the displays become drastically light and flexible. However, it is difficult to obtain the substrate materials simultaneously possessing excellent combined properties, *i.e.*, optical transparency, heat resistance, dimensional stability against thermal cycles undergoing in the device fabrication process, flexibility, and processability. The most effective strategy for completely erasing the coloration is to inhibit the CT interactions by using non-aromatic (cycloaliphatic) monomers either in tetracarboxylic dianhydride or diamine components. For this purpose, we previously investigated the steric structures of hydrogenated pyromellitic dianhydride isomers, *i.e.*, 1*S*,2*R*,4*S*,5*R*-cyclohexanetetracarboxylic dianhydride (H-PMDA) (Uchida *et al.*, 2003) and 1*R*,2*S*,4*S*,5*R*-cyclohexanetetracarboxylic dianhydride (H"-PMDA) (Uchida *et al.*, 2012). H"-PMDA showed much higher reactivity with diamines than H-PMDA and provided highly flexible colourless PI films with significantly improved solution-processability while keeping very high *T*_g_s (Hasegawa *et al.*, 2007, 2013). The results are based on a peculiar steric structure of H"-PMDA. Unfortunately, neither H-PMDA nor H"-PMDA led to PI films with low coefficients of thermal expansion (CTE) required for the excellent dimensional stability, probably owing to their non-linear/non-planar steric structures. An additional H-PMDA isomer, *i.e.*, 1*S*,2*S*,4*R*,5*R*-cyclohexanetetracarboxylic dianhydride (H'-PMDA) can be expected to derive a novel low-CTE colourless PI system. The present work reports a crystal structure of a hydrolyzed compound of H'-PMDA.

### 2. Experimental

The title compound, (I), was synthesized as follows. Pyromellitic dianhydride was first hydrolyzed with a NaOH aqueous solution. The pyromellitic acid tetrasodium salt formed was hydrogenated in a high-pressure hydrogen atmosphere at 160 °C in the presence of a ruthenium catalyst. After hydrogenation was completed, the solution was additionally heated at a precisely controlled temperature for several hours, and cooled to room temperature. The solution was neutralized by slowly adding conc. HCl. The white precipitate formed was collected by filtration, recrystallized from water, and dried in vacuum at 80 °C for 5 h to obtain crystals of (I) suitable for X-ray analysis.

### 3. Refinement

All H atoms were observable in a difference Fourier map. H atoms on O atoms were refined freely [O—H = 0.81 (3) and 0.94 (3) Å]. Other H atoms were placed in calculated positions with C—H = 0.97–0.98 Å, and allowed to ride on their carrier atoms, with *U*_iso_(H) = 1.2*U*_eq_(C).

### Crystal data

**Table:**

C_10_H_12_O_8_	*D*_x_ = 1.502 Mg m^−^^3^
*M**_r_* = 260.20	Mo *K*α radiation, λ = 0.71073 Å
Trigonal, *R*3	Cell parameters from 2340 reflections
*a* = 17.6970 (6) Å	θ = 2.3–30.0°
*c* = 9.5455 (6) Å	µ = 0.13 mm^−^^1^
*V* = 2589.0 (2) Å^3^	*T* = 298 K
*Z* = 9	Block, colourless
*F*(000) = 1224	0.33 × 0.26 × 0.26 mm

### Data collection

**Table:**

Bruker APEXII CCD area-detector diffractometer	1388 reflections with *I* > 2σ(*I*)
Radiation source: fine-focus sealed tube	*R*_int_ = 0.019
φ and ω scans	θ_max_ = 30.0°, θ_min_ = 2.3°
Absorption correction: multi-scan (*SADABS*; Sheldrick, 1996)	*h* = −24→21
*T*_min_ = 0.891, *T*_max_ = 0.966	*k* = −20→24
6439 measured reflections	*l* = −13→11
1653 independent reflections	

### Refinement

**Table:**

Refinement on *F*^2^	0 restraints
Least-squares matrix: full	Hydrogen site location: mixed
*R*[*F*^2^ > 2σ(*F*^2^)] = 0.041	H atoms treated by a mixture of independent and constrained refinement
*wR*(*F*^2^) = 0.119	*w* = 1/[σ^2^(*F*_o_^2^) + (0.0607*P*)^2^ + 1.9122*P*] where *P* = (*F*_o_^2^ + 2*F*_c_^2^)/3
*S* = 1.06	(Δ/σ)_max_ < 0.001
1653 reflections	Δρ_max_ = 0.38 e Å^−^^3^
90 parameters	Δρ_min_ = −0.21 e Å^−^^3^

### Special details

**Table:**

Geometry. All e.s.d.'s (except the e.s.d. in the dihedral angle between two l.s. planes) are estimated using the full covariance matrix. The cell e.s.d.'s are taken into account individually in the estimation of e.s.d.'s in distances, angles and torsion angles; correlations between e.s.d.'s in cell parameters are only used when they are defined by crystal symmetry. An approximate (isotropic) treatment of cell e.s.d.'s is used for estimating e.s.d.'s involving l.s. planes.

### Fractional atomic coordinates and isotropic or equivalent isotropic displacement parameters (Å^2^)

**Table:**

	*x*	*y*	*z*	*U*_iso_*/*U*_eq_	
C1	0.51496 (8)	0.07671 (7)	−0.08154 (11)	0.0279 (3)	
H1A	0.4929	0.1116	−0.1242	0.034*	
H1B	0.5770	0.1045	−0.1011	0.034*	
C2	0.50063 (7)	0.07293 (7)	0.07787 (11)	0.0233 (2)	
H2	0.4383	0.0484	0.0969	0.028*	
C3	0.53165 (7)	0.01523 (7)	0.14585 (11)	0.0242 (2)	
H3	0.5944	0.0414	0.1285	0.029*	
C4	0.55013 (8)	0.16503 (7)	0.13456 (12)	0.0283 (3)	
C5	0.51702 (8)	0.00882 (8)	0.30236 (12)	0.0267 (3)	
O1	0.62253 (6)	0.19739 (7)	0.18327 (13)	0.0471 (3)	
H4	0.5352 (17)	0.2558 (18)	0.151 (3)	0.083 (8)*	
O2	0.50596 (8)	0.20615 (8)	0.12244 (15)	0.0543 (3)	
O3	0.46919 (7)	0.03210 (7)	0.35746 (9)	0.0380 (3)	
O4	0.55716 (7)	−0.02417 (7)	0.37025 (10)	0.0400 (3)	
H5	0.5485 (19)	−0.0245 (19)	0.467 (3)	0.110 (10)*	

### Atomic displacement parameters (Å^2^)

**Table:**

	*U*^11^	*U*^22^	*U*^33^	*U*^12^	*U*^13^	*U*^23^
C1	0.0389 (6)	0.0247 (5)	0.0218 (5)	0.0171 (5)	0.0021 (4)	0.0033 (4)
C2	0.0259 (5)	0.0227 (5)	0.0214 (5)	0.0123 (4)	0.0009 (4)	0.0002 (4)
C3	0.0274 (5)	0.0268 (5)	0.0198 (5)	0.0147 (4)	0.0014 (4)	0.0015 (4)
C4	0.0309 (6)	0.0246 (5)	0.0267 (5)	0.0119 (5)	0.0041 (4)	0.0008 (4)
C5	0.0310 (6)	0.0282 (5)	0.0214 (5)	0.0153 (5)	−0.0010 (4)	0.0009 (4)
O1	0.0302 (5)	0.0342 (5)	0.0672 (8)	0.0088 (4)	−0.0056 (5)	−0.0095 (5)
O2	0.0559 (7)	0.0333 (5)	0.0820 (9)	0.0285 (5)	−0.0212 (6)	−0.0187 (5)
O3	0.0517 (6)	0.0540 (6)	0.0237 (4)	0.0380 (5)	0.0042 (4)	0.0031 (4)
O4	0.0531 (6)	0.0611 (7)	0.0238 (4)	0.0420 (6)	0.0015 (4)	0.0059 (4)

### Geometric parameters (Å, º)

**Table:**

C1—C3^i^	1.5370 (16)	C3—H3	0.9800
C1—C2	1.5386 (15)	C4—O1	1.2049 (16)
C1—H1A	0.9700	C4—O2	1.3126 (16)
C1—H1B	0.9700	C5—O3	1.2295 (15)
C2—C4	1.5130 (15)	C5—O4	1.2961 (14)
C2—C3	1.5251 (15)	O2—H4	0.81 (3)
C2—H2	0.9800	O4—H5	0.94 (3)
C3—C5	1.5108 (15)		
			
C3^i^—C1—C2	111.06 (9)	C5—C3—C1^i^	109.53 (9)
C3^i^—C1—H1A	109.4	C2—C3—C1^i^	110.89 (9)
C2—C1—H1A	109.4	C5—C3—H3	108.3
C3^i^—C1—H1B	109.4	C2—C3—H3	108.3
C2—C1—H1B	109.4	C1^i^—C3—H3	108.3
H1A—C1—H1B	108.0	O1—C4—O2	123.84 (12)
C4—C2—C3	111.15 (9)	O1—C4—C2	123.74 (11)
C4—C2—C1	108.23 (9)	O2—C4—C2	112.41 (11)
C3—C2—C1	110.07 (9)	O3—C5—O4	124.14 (11)
C4—C2—H2	109.1	O3—C5—C3	121.25 (10)
C3—C2—H2	109.1	O4—C5—C3	114.60 (10)
C1—C2—H2	109.1	C4—O2—H4	109.5 (18)
C5—C3—C2	111.43 (9)	C5—O4—H5	111.8 (17)
			
C3^i^—C1—C2—C4	178.45 (9)	C1—C2—C4—O1	−95.39 (14)
C3^i^—C1—C2—C3	56.81 (13)	C3—C2—C4—O2	−155.53 (11)
C4—C2—C3—C5	61.11 (12)	C1—C2—C4—O2	83.49 (13)
C1—C2—C3—C5	−179.00 (9)	C2—C3—C5—O3	14.47 (16)
C4—C2—C3—C1^i^	−176.60 (9)	C1^i^—C3—C5—O3	−108.61 (13)
C1—C2—C3—C1^i^	−56.70 (13)	C2—C3—C5—O4	−166.81 (10)
C3—C2—C4—O1	25.59 (16)	C1^i^—C3—C5—O4	70.12 (13)

Symmetry code: (i) −*x*+1, −*y*, −*z*.

### Hydrogen-bond geometry (Å, º)

**Table:**

*D*—H···*A*	*D*—H	H···*A*	*D*···*A*	*D*—H···*A*
O2—H4···O1^ii^	0.81 (3)	1.93 (3)	2.705 (2)	160 (3)
O4—H5···O3^iii^	0.94 (3)	1.70 (3)	2.632 (1)	176 (3)

Symmetry codes: (ii) −*x*+*y*+1, −*x*+1, *z*; (iii) −*x*+1, −*y*, −*z*+1.
